# Education and National Morality

**Published:** 1917-09-15

**Authors:** 


### SOME PROBLEMS OF TO-DAY.

### VI. Education and National Morality.

If the minimum proposal for continued education comes shortly into operation as regards girls, we shall find ourselves face to face with a new problem, or, more correctly, with a new aspect of a very ancient problem. Eight hours a week for forty weeks in the year will involve some readjustment in industries, and also in domestic service, if the employer's time is to be used, and if girls are to continue their studies till the age of eighteen. As a fact, the sacrifice made by the employer will be a good deal discounted if it proves true that increased efficiency at work is a direct and prompt result in a majority of cases. But so far nothing seems to have been said about the young employees of "the oldest trade in the world," though this point cannot be called an unimportant one. Indeed, in several ways its importance has lately been emphasised. For instance, all authorities in venereal disease agree in stating that the danger of infection is nowhere so serious as among young girls who have newly taken to immoral lives, older women frequently proving immune. Also the younger girls who have been led astray a first time far more frequently become mothers than older ones who have learned how to avoid the consequences of their actions. It is among the children of these girls that infant mortality is so abnormally high—twice, if not three times that of fathered babies—and equally among them the survival of the unfit constitutes the most serious problems. The feeble-minded, the criminal, the ill-developed, who burden the finances of the country, are recruited numerously enough from these unwelcomed gifts of Heaven. Do not let us forget that one influence of that raising of moral tone and public opinion which has been the outcome of so much devoted labour has been to make the stigma of illegitimacy cast over young lives a shadow which did not fall till the brighter light was kindled. The bitterness of this Ishmael position has hardened many a young heart into enmity against society. A strangely pathetic tale was told some years ago of one such child, who when boarded out in a country village was invited to join a birthday party, and learnt for the first time what a birthday meant, with a sudden revelation of all she had lost. It is pleasant to add that the Guardians were human enough to ordain a birthday for this forlorn child. To them also the trivial incident was perhaps a revelation.

Hitherto, of course, any girl leaving school has been able, if desirous to do so, to pursue an immoral life without let or hindrance unless she contravened certain provisions of the Children Act within recent years. Parental authority, once she became a wage-earner, had no force beyond moral suasion and the sacred influences of example and of affection. Definite rescue-work, advising and helping her to change her mode of life, and definite preventive work, if brought to bear upon her, would come through the unselfish labours of good women in the name of religion. For obviously it is only in the name of religion that any successful appeal can be made to a young woman who deliberately chooses the good pay and luxurious surroundings obtainable during her best years. "What can you offer me that is better than this?" is often the frank answer given by the inmate of a well-appointed flat. And such girls are often very young. In a particularly painful recent case the flat was managed by a girl of seventeen, who was brought to book because she had abducted one of only fourteen from a provincial town, and this child asserted in court that many schoolgirls in that town were making good incomes by vicious courses. To offer such young persons altruistic arguments of citizenship is obviously futile; nothing less than conversion, a turning round of the whole soul and mind by belief with which the deepest and strongest emotion mingles, can now avail. It is quite otherwise when the character is being built up by education, a training which upholds noblest ideals for the simplest life can then form bulwarks not easily sapped by idleness and self-indulgence, and finally broken down by temptation.

But the power of evil is strong, and wants nothing less than all the available force of goodness to resist it. And here we come to the kernel of our question. The State has declared healthy and upright citizens to be its most precious asset. It has asserted this especially of the children, as its prime need in quantity and quality. And the State declares itself Christian. Its gold coinage bears the image of the saintly dragon-slayer, its National Anthem is a prayer to Almighty God, its highest military award is a cross. Its laws have been headed since Alfred placed them there by the Ten Commandments, and one of those prohibitions uncompromisingly forbids "the oldest trade in the world." Whether, therefore, we view the situation on the highest moral ground or on the lowest economic one, it is certain that the waste of life, of health, and of national resources is criminal and deplorable. Even those who have been content to say that "such things always have been and always will be" must feel a gulf yawning at their feet when they face the unthinkable position of girls coming to school from and returning again to a house of ill-fame. Here on the other hand, is a real opportunity for those who believe that social reconstruction can mean social regeneration, and who are honestly willing to work for it. Let them urge that a better occupation of time, in recreation as well as in employment and in lessons, shall be taught to the working man's child, and also made possible. Whether definite sex-instruction should be given or not is another—and in fact a subordinate—consideration, for what is far more needed is a sense of responsibility on the part alike of parents, teachers, employers, the community generally, and the adolescents themselves for the protection and training of these immature citizens. That responsibility the Church of England long ago laid on the lay people of the country when she provided that for every child a small working committee of three persons, called by the old name of god-parents, should assist and supplement the training of parents. These persons were to see that the child learnt the principles of the faith, and in due time assumed responsibility and received the Church's benediction and a Divine gift of spiritual strength. They were also to see to it that the child was "virtuously brought up." Only the task of teaching was imposed upon the clergy. Whether we will it or no, this responsibility for the adolescents has now been assumed afresh by the community, and distinctly expressed in the decision for continued education. Shall we have the courage to be logical?

If so, the State will have something to say to men who are guilty of what Mrs. Bramwell Booth lately called our worst national sin, the theft of a woman's best possession, the casting upon her and upon his country of his own lawful burden, the maintenance of his own child. It will not leave to the deserted girl the task of finding him and trying to bring him to book, with the temptation naturally involved to herself cast off the burden. It will also have courage to apply some sensible coercive measures to some extremely naughty little girls.

☛ Previous articles appeared March 31, April 7, May 5, 19, and 26.

